# Dental caries prevalence, oral health knowledge and practice among indigenous Chepang school children of Nepal

**DOI:** 10.1186/1472-6831-13-20

**Published:** 2013-05-14

**Authors:** Lonim Prasai Dixit, Ajay Shakya, Manash Shrestha, Ayush Shrestha

**Affiliations:** 1 Department of Community Dentistry, Peoples Dental College and Hospital (PDCH), Kathmandu, Nepal; 2 Dental Surgeon, PDCH, Kathmandu, Nepal

**Keywords:** Dental caries, School children, Oral hygiene

## Abstract

**Background:**

Chepang communities are one of the most deprived ethnic communities in Nepal. According to the National Pathfinder Survey, dental caries is a highly prevalent childhood disease in Nepal. There is no data concerning the prevalence of caries along with knowledge, attitude and oral hygiene practices among Chepang schoolchildren. The objectives of this study were to 1) record the prevalence of dental caries 2) report experience of dental pain 3) evaluate knowledge, attitude and preventive practices on oral health of primary Chepang schoolchildren.

**Method:**

A cross sectional epidemiological study was conducted in 5 government Primary schools of remote Chandibhanjyang Village Development Committee (VDC) in Chitwan district. Ethical approval was taken from the Institutional Review Board within the Research Department of the Institute of Medicine (IOM) Tribhuvan University. Consent was obtained from parents for conducting clinical examination and administrating questionnaire. Permission was taken from the school principal in all schools. Data was collected using a pretested questionnaire on 131 schoolchildren aged 8-16-year- olds attending Grade 3–5. Clinical examination was conducted on 361 school children aged 5–16 –year-olds attending grade 1–5. Criteria set by the World Health Organization (1997) was used for caries diagnosis. The questionnaires, originally constructed in English and translated into Nepali were administered to the schoolchildren by the researchers. SPSS 11software was used for data analysis.

**Results:**

Caries prevalence for 5–6 –year-old was above the goals recommended by WHO and Federation of Dentistry international (FDI) of less than 50% caries free children. Caries prevalence in 5-6-year-olds was 52% and 12-13-year-olds was 41%. The mean dmft/DMFT score of 5–6 –year-olds and 12 -13-year -olds was 1.59, 0.31 and 0.52, 0.84 respectively. The DMFT scores increased with age and the d/D component constituted almost the entire dmft/DMFT index. About 31% of 8-16-year-olds school children who participated in the survey reported having suffered from oral pain. Further, the need for treatment of decayed teeth was reported at 100%. About 76% children perceived teeth as an important component of general health and 75% reported it was required to eat. A total 93% children never visited a dentist or a health care service. Out of 56% children reporting cleaning their teeth daily, only 24% reported brushing their teeth twice daily. About 86% of the children reported using toothbrush and toothpaste to clean their teeth. Although 61% children reported to have received oral health education, 82% children did not know about fluoride and its benefit on dental health. About 50% children reported bacteria as the main cause of tooth decay and 23% as not brushing teeth for gingivitis. Frequency of sugar exposure was low; 75% of children reported eating sugar rich food once daily.

**Conclusions:**

Caries prevalence of 5–6 –year- old Chepang school children is above the recommended target set by FDI/WHO. The study reported 31% schoolchildren aged 8-16-year old suffered oral pain and decayed component constituted almost the entire dmft/DMFT index. The brushing habit was reportedly low with only 24% of the children brushing twice daily. A nationwide scientifically proven, cost effective school based interventions is needed for prevention and control of caries in schoolchildren in Nepal.

## Background

Dental caries is considered a major public health problem globally due to its high prevalence and significant social impact. World Health Organization reports 60-90% of schoolchildren worldwide have experienced caries, with the disease being most prevalent in Asian and Latin American countries [1]. Nearly 41 percent of the total population in Nepal is under the age of 16 years, and of the total school age children, 87 percent are enrolled in schools. The 2004 National Pathfinder Survey shows that 58% of 5–6 –year- old schoolchildren suffer from dental caries [2]. With the caries prevalence of 58%, dental caries is more prevalent than malnutrition that affects 49% of child population [3]. The Survey reported pain and discomfort due to untreated dental caries in 18% of 5–6 –year-olds and 64% in older adults. Adolescent school children reported inability to eat followed by inability to speak. Studies have reported missed school hours, toothache and several impairments of daily life activities associated with a high decayed component in both primary and permanent dentition [4]. Similar findings have been reported in Brazilian preschool children and in a school survey of American native children [5,6]. A study in the Philippines had previously reported significant association between untreated dental caries and low Body Mass Index [7]. Interplay of socioeconomic, environmental and behavioral factors, constitute the social determinants of health, are driving the epidemics of communicable and noncommunicable diseases [8]. In Low-Income Countries (LICs), health related behaviors among children are low [9]. Nutritional transition with easy access to refined carbohydrates, low use of fluoridated toothpaste and irregular tooth brushing habits lead to increasing trend in dental caries in developing countries. Schools provide the ideal setting to reach millions of children and ensure strong foundations for a healthy life at an early stage. The principles outlined in Ottawa Charter focus on healthy public policy and strengthening skills and practices. Focusing efforts in practical school based health activities will help to reduce inequalities in health. There is no national data concerning the prevalence of dental caries among Chepang schoolchildren in Nepal. This is the only epidemiological study conducted in schoolchildren of Chepang communities residing in remote hilly region of Chitwan district in Nepal. Although Chitwan is considered a relatively developed district in Nepal, the Chepang communities face many development issues and belong to one of the most disadvantaged ethnic communities. Out of approximately 52,000 Chepang population, majority live in the poorest of conditions in remote hills. Many school children living in remote areas of Nepal including Chepang lack hygiene practices that could prevent number of chronic diseases. The study aims to highlight the need for an evidence-based cost effective integrated hygiene and sanitation facilities in schools. The objectives of this study were to 1) record the prevalence of dental caries, 2) report experience of dental pain 3) evaluate knowledge, attitude and preventive practices on oral health of 5–14 year -old Chepang school children.

## Methods

A cross sectional epidemiological study was conducted in 5 government schools of remote Chandibhanjyang VDC in Chitwan district. Ethical approval was received from the Institutional Review Board at the Institute of Medicine. All five primary schools in the working area of the Department of Community Dentistry, Peoples Dental College and Hospital was surveyed. A study protocol was presented and ethical approval was received from the Institutional Review Board within the Research Department of the Institute of Medicine (IOM) Tribhuvan University. Consent was obtained from parents who had been informed by the school management prior to data collection. Permission to conduct the research as a part of the school based oral health project was taken from the school principal of each school. Convenient sampling was used as the research was conducted in schools that were part of the school health project of the Department of Community Dentistry, Peoples Dental College and Hospital. The researchers were trained by the Chief researcher on the methodology proposed by WHO, 1997. The researchers had experience collecting data on dental caries using the WHO, 1997 methodology in school camp conducted each month by the Department. Clinical examination was conducted on 361schoolchildren 5- 16-year-olds attending Grade 1–5. The status of dental caries was recorded using the guideline of WHO Oral Health Survey Basic Methods 1997 [10]. All examinations were carried out in well-lit classrooms. Data on knowledge and practice was collected using structured questionnaires on 131schoolchildren. Questionnaire was administered to all children aged 8-16- year-olds attending Grade 3–5 who were willing to be a part of the study. Children in grade 1 and 2 were not included in the questionnaire survey. The questionnaire was pretested in similar survey conducted prior to the present study in similar environment. Structured questionnaire was originally constructed in English and translated in Nepali language for local use. The researchers administered the questionnaire and the teachers assisted the researchers on administrative matters and did not interfere with administration of the questionnaire. The respondents were asked questions on basic knowledge on oral heath, attitude and oral hygiene practices. The questions on oral health knowledge comprised of causes of tooth decay, what tooth decay is, causes of gingivitis, gum swelling, reason for cleaning teeth, what fluoride is, fluoride content in toothpaste, use of fluoride, its effect on dentition and importance of teeth. Questions were asked on brushing habit, aids used for brushing, brushing frequency, rinsing habit in order to assess practice on oral health. The respondents were also given questionnaires on experience of dental pain, any prior information received on oral health and sources of information if received. SPSS 11software was used for data analysis.

## Results

The study population consists of 361 school age children with age group from 5–16 years. A total of 178 children were boys and 183 were girls. Structured questionnaire was administered to 8-16-year-olds 131 school children (60 boys and 71 girls) attending grades 3–5. About 31% of 8-16-year-olds who participated in the survey reported having suffered from oral pain (Table 1). Caries prevalence and mean dmft/DMFT score of 5-6-year-olds and 12 -13-year-olds was 52%, 1.59 and 41%, 0.84 respectively (Table 2, 3). An increasing trend in DMFT was seen with increasing age (Table 3). Only 56% children reported having cleaned their teeth daily but 80% children reported to rinse mouth with water after meals (Table 4). Out of 56% children reporting cleaning their teeth daily, only 24% reported brushing their teeth twice daily. However, 86% of the children reported using toothbrush and toothpaste to clean their teeth. When asked about knowledge, 40% children reported having brushed their teeth for keeping it clean and 25% children to make teeth and gums strong (Table 5). About 76% children perceived teeth an important component of general health and 75% reported it was required to eat. The study reported low dental visit with 93% of children having never visited a dentist or a health care service. Although 61% children reported receiving oral health education, 82% did not know about fluoride and its benefit on dental health. About 50% children reported bacteria as the main cause of tooth decay and 23% as not brushing teeth for gingivitis. Among the 361 school children clinically examined, overall dental caries prevalence was 45%. Further, the need for treatment of decayed teeth was reported at 100%.

**Table 1:** Reported percentage experience of tooth pain in children by gender

	**Total number**	**Yes pain**	**No pain**	**Prevalence(%)**
Boys	**60**	**21**	**39**	**35**
Girls	**71**	**20**	**51**	**28**
	**131**	**41**	**90**	**31**

P value: non significant.

**Table 2:** Prevalence proportion (%) of dental caries by gender and age groups

		**Over all**		**5-6 years**		**12-13 years**	
	**Total number (n)**	**Caries present (n)**	**Overall prevalence (%)**	**Caries present (n)**	**Total prevalence (%)**	**Caries present (n)**	**Total prevalence (%)**
Boys	**178**	**83**	**55**	**19**	**61.3**	**8**	**32**
Girls	**183**	**78**	**44**	**11**	**40.7**	**15**	**48.4**
Total	**361**	**161**	**45**	**30**	**52**	**23**	**41**

P value: non significant.

**Table 3:** Mean caries indices in different age groups examined

**Age group**	**Mean dft**	**Mean DMFT**
5-6 years	1.59	0.31
7-11 years	1.11	0.51
12-13 years	0.52	0.84
14-16 years		0.70
Total	1.15	0.57

**Table 4:** Percentage of total subjects on preventive practice on oral health

	**Yes**	**Total number**	**Percentage**
Cleans teeth daily	**73**	**131**	**56**
Rinses mouth daily	**105**	**131**	**80**
Brushes twice daily	**31**	**131**	**24**
Use of toothbrush and toothpaste	**113**	**131**	**87**

**Table 5:** Percentage of total subjects with knowledge on oral health

	**Yes**	**Total number**	**Percentage**
Sugar causes caries	**66**	**131**	**50**
Not brushing causes gingivitis	**30**	**131**	**23**
Bacteria causes gingivitis	**17**	**131**	**13**
Benefits of fluoride on dental health	**23**	**131**	**18**

## Discussion and conclusions

In Nepal, there is scarce data on the oral health status of children attending public schools. The first and only national oral health survey was conducted in 2004. Although the present study is not based on representative sample of public school children in Nepal (as it uses convenient sampling) it does give an insight into the caries prevalence, oral health knowledge and preventive practice in rural public schools. There are many communities in Nepal like the Chepang, where children only have access to public schools. A study conducted in Central and Western Nepal among 5–6 year old schoolchildren, reported 67% of children were affected by dental caries [11]. In the present study, caries prevalence in 5-6- year-old was 52%, which is in line with the national pathfinder survey conducted in 2004 where caries was reported to affect 58% of 5-6- year-olds. The dental caries prevalence among 5–6 –year-old Chepang schoolchildren is above the recommended target of WHO and Federation of Dentistry International of having less than 50% caries free children by 2000 [12]. Experience of oral pain was high with 31% of school children reporting experiencing dental pain. Another study conducted in Nepal among 9–11 years old schoolchildren had reported that 45% of children suffered from tooth pain [13]. In the same study, it was reported that 93% of children had never visited a dentist and the decayed component constituted almost entire dmft/DMFT index. Low dental visit and 100% untreated caries could be due to lack of access to affordable health care services. Similar finding of high prevalence of untreated dental caries was reported in studies in schoolchildren who had limited access to dental care [14-16]. Rinsing mouth with water is a common practice in Nepali culture. The study reported 80% children rinsing mouth with water. The most common aid used for maintaining oral hygiene was toothbrush and toothpaste. Tooth brushing once a day is a norm in Nepal. Only 56% school children brushed their teeth daily. Brushing twice daily with fluoridated toothpaste is a recommended practice for good oral health [17]. Although Nepal is undergoing nutrition transition phase, the Chepang children do not seem to be affected due to lack of access to refined carbohydrate and sugar rich foods and drinks. The frequency of consuming sugar rich foods and aerated drinks was very low. Only 75% children consumed sugar rich foods once daily. About 61% of children reported to have received oral health education. However, knowledge on fluoride and its effect on oral health was low. About 82% children did not know about fluoride and its benefits on dentition. A considerable amount of school children had never been to a dentist nor had access to preventive hygiene practices. WHO has recommended for public health efforts to make fluoridated toothpaste affordable in developing countries [18]. Intersectoral coordination with education, government sectors and development of public health policy have profound effect in improving the health of the community people [19]. Schools provide an ideal setting for providing oral health education at early stage. However education itself is not enough to bring tangible changes in behavior change. Another opportunity to utilize the school setting would be to ensure lifelong skills such as school based tooth brushing and hand washing. In a country like Nepal, where health care facilities are not accessible and affordable to many communities including the Chepang, health promotion and prevention plays a very important role. Schools are ideal setting for health promotion. A basic prioritized action plan using proven models and approaches can tangibly accelerate improvements in school health in Nepal reaching millions of school children.

## Competing interest

The authors of this article declare that they have no competing interests.

## Authors’ contribution

LPD and AS have played a role in design of the study, data analysis and interpretation. LPD has acquired funding for the project and research and been involved in drafting and revising the manuscript. MS and AS have participated in data collection, data analysis and also contributed to the design of the study. All authors read and approved the final manuscript.

## Pre-publication history

The pre-publication history for this paper can be accessed here:

http://www.biomedcentral.com/1472-6831/13/20/prepub
